# High-yawning male rats are more sexually motivated when exposed to sexually receptive female rats in a multichoice sexual paradigm

**DOI:** 10.1016/j.ibneur.2026.04.009

**Published:** 2026-04-21

**Authors:** Carmen Cortes, Alfonso Mora, Juan M. Ibarra-Hernández, Araceli Ugarte, Jose R. Eguibar

**Affiliations:** a Institute of Physiology, Benemérita Universidad Autónoma de Puebla. Col. Jardines de San Manuel, Pue. C.P, Av. 14 Sur No. 6301, Puebla 72570, Mexico; b Department of Physiology, Faculty of Medicine, Universidad Autónoma de Nuevo León. Av. Doctor José Eleuterio González 235, Mitra Centro. C.P, Monterrey, N.L 64460, Mexico; c Internationalization Office, Benemérita Universidad Autónoma de Puebla. Torre de Gestión Académica y Servicios Administrativos, 3er piso. Col. Jardines de San Manuel, Pue C.P, Puebla 72570, Mexico

**Keywords:** Phenotype, Mating, Motivation, Calls, Ultrasonic vocalizations, Communication

## Abstract

During mating, several sensory cues are involved in the sexual interaction process, among which the emission of 50 kHz ultrasonic vocalizations (USVs) plays a key role. We have established two sublines from Sprague–Dawley (SD) rats whose spontaneous yawning frequency differs: high-yawning (HY) with a mean of 20 yawns/h and low-yawning (LY) with a mean of only 2 yawns/h. The HY male rats had more spontaneous penile erections, and when they mated with ovariectomized females primed by sequential administration of estradiol benzoate followed by progesterone for 44 h, HY males had more sexual bouts and longer interintromission intervals that delayed ejaculation during paced mating. During mating, male and female rats emitted 50 kHz ultrasonic vocalizations (USVs), which are a sexual incentive, and these calls promoted sexual interaction.

All the tested rats were maintained under standard conditions, and the USVs were recorded with a plug-and-play ultrasound microphone and analyzed using UltraVox XT software. When the rats were evaluated in a four-arm maze paradigm, three arms contained sexually experienced HY, LY or SD males, and the other arm was an empty compartment. Ovariectomized estrous-induced females of each phenotype were introduced and allowed to freely explore the maze for 5 min. All the male groups of rats emitted significantly more USVs when exposed to a receptive female rat than when they were not exposed. Importantly, compared with LY and SD males, HY male had more frequency-modulated and flat calls with shorter latencies, and HY males were significantly preferred by SD and LY estrous-induced females. Overall, HY males had greater sexual motivation associated with a more positive appetitive state.

## Highlights

• 1.- High-yawning male rats emitted more vocalizations because of higher motivation status.
• 2.- High-yawning male rats had a higher degree of positive emotional arousal.
• 3.- High-yawning male rats emitted more frequency-modulated and flat vocalizations.
• 4.- Low-yawing males were less sexually motivated.

## 1 Introduction

Emission of ultrasonic vocalizations (USVs) plays several behavioral roles in interindividual communication and social engagement, which are critical for animal social survival (Simola and Brudzynski, 2018). In fact, the emission of USVs plays a key role in the formation of social bonds, social cooperation, and maintenance of affiliative processes within a social group (Simola and Brudzynski, 2018, Tyack, 2008). Rats emit USVs in the 22–50 kHz range. These calls have been extensively studied, and they have been shown to carry semiotic value, have symbolic reference, and can change the behavior of the signal recipient (Demaestri et al., 2019, Tyack, 2008). Additionally, there is evidence indicating that 50 kHz USVs are indicative of a positive affective state associated with anticipation of reward and/or positive social-emotional contexts (Wöhr and Schwarting, 2007).

In rats, USVs are useful tools for determining emotional states and motivation. USVs in rats are valence specific because rats emit 22-kHz vocalizations during aversive states and 50-kHz vocalizations during appetitive states (Brudzynski, 2021, Burgdorf et al., 2020). Therefore, 50-kHz USVs are a measure of many aspects of emotional and motivated behavior, and it must be considered that USVs convey biologically significant information to conspecifics. In fact, measuring the emission of 50-kHz USVs is a way to evaluate positive emotional states, and these USVs include heterogeneous calls of short duration and high frequency (approximately 50 kHz). Moreover, these calls have frequency-modulated (FM), flat and trill subtypes. In fact, 50-kHz FM ultrasonic vocalizations reflect positive emotional arousal; thus, recording these calls is a reliable tool for evaluating this condition (Brudzynski, 2021, Burgdorf et al., 2011, Burgdorf et al., 2020).

Importantly, the ascending mesolimbic dopaminergic system mediates appetitive and rewarding emotional arousal through the release of dopamine in the nucleus accumbens (NAcc), particularly in the shell region (Willhuhn et al., 2014). Therefore, the limbic dopaminergic system is the substrate for the initiation of 50-kHz USV emission (Brudzynski, 2012). One important appetitive behavior is sexual behavior, and its display clearly induces a positive rewarding state. For example, ejaculations induce a conditioning mate place preference, and this state is strongly rewarding and similar to that of an addictive drug such as a dopamine-releasing or opiate drug (Agmo and Paredes, 1988).

In our laboratory at the Institute of Physiology, Benemérita Universidad Autónoma de Puebla, we selectively inbred two sublines of Sprague–Dawley (SD) rats, whose spontaneous yawning frequency differs (Urbá-Holmgren et al., 1990). The high-yawning (HY) subline has a yawning frequency of 20 yawns/h, and the low-yawning subline has a yawning frequency of only 2 times/h (Urbá-Holmgren et al., 1990, Holmgren et al., 1985). Subsequent studies have demonstrated that compared with male LY rats, male HY rats have a different copulatory pattern because they display more copulatory bouts, with longer interintromission intervals that delay ejaculation, when they are copulated with ovariectomized SD rats with estrous induced by sequential administration of estradiol benzoate followed by progesterone (Eguibar et al., 2016). Additionally, spontaneous yawning and penile erections are time correlated in a narrow three-minute time window when penile erection occurs, and this is also the case when yawning and penile erections are induced by systemic administration of D2-like dopaminergic agonists such as apomorphine and quinpirole, with these responses being greater in HY rats than in LY rats (Eguibar et al., 2003, Urbá-Holmgren et al., 1993). In an analogous way, the central administration of oxytocin also induces higher yawning and penile erection frequencies in adult male HY rats than in adult male LY rats (Eguibar et al., 2015).

The aim of this study was to analyze the different 50-kHz ultrasonic vocalizations emitted by high-yawning, low-yawning and Sprague–Dawley males when they interact with ovariectomized females in which estrous was induced by the sequential injection of estradiol benzoate followed by progesterone in a four-arm maze paradigm.

## 2 Materials and methods

The present study included 24 rats—eight from each yawning subline and eight outbred Sprague–Dawley male rats weighing 305 ± 15 g at the start of the recording sessions at 90 ± 2 postnatal days (PND). The rats were housed in Plexiglas cages (46 × 32 × 20 cm) in groups of 2–3 rats per cage in an experimental colony room with controlled temperature conditions at 22 ± 2 °C, a relative humidity between 35%–50%, and a 14:10 light–dark cycle (lights on 22:00). Balanced rodent pellets (5008; Purina Mills, USA) and purified Ciel™ water (Coca Cola Co., México) were always available.

All procedures described in this study were performed in compliance with the Mexican official standard technical specifications for the production, care and use of laboratory animals (NOM-062-ZOO-1999) and with the Seventh Title of the Regulations of the General Health Law on Health Research of the Mexican government, as well as the Guide for the Care and Use of Laboratory Animals of the Institute for Laboratory Animal Research and the National Research Council of the National Academies (National Research Council, 2011). ARRIVE guidelines 2.0 for animal research were followed throughout the study (Percie du Sert et al., 2020). The Institutional Committee for the Care and Use of Laboratory Animals of the Benemérita Universidad Autónoma de Puebla (CICUAL-Proyecto BUAP- 15755) approved all the animal procedures.

### 2.1 Surgery

#### 2.1.1 Ovariectomy

The rats were anesthetized with an intraperitoneal injection of ketamine (75 mg/kg) and xylazine (5 mg/kg). The rats were placed on a heated surgical platform (Conair, USA) to maintain their core body temperature at 37 ± 0.2 °C throughout the procedure. The abdominal region was shaved using an electric razor (Oster, USA) and antisepticized with 10% povidone-iodine (Farmacéutica Altamirano, México), and a 1 cm midline incision was made using a scalpel just below the line of the last costal body. A small lateral abdominal incision was made to gently exteriorize the ovary, and the oviduct was ligated with two absorbable sutures, after which the ovary was removed. The same procedure was performed on the contralateral side. The incisions were closed with interrupted nonabsorbable sutures, and the animals were returned to their home cages for recovery. Postoperative care included subcutaneous administration of the analgesic flunixin meglumine (Napzin®, 2 mg/kg) and the antibiotic enrofloxacin (Enroxil™ 5%, 0.15 mg/kg) once daily for five consecutive days under the supervision of a certified veterinary physician.

### 2.2 Procedures

All male rats obtained sexual experience through four consecutive sessions of sexual mating every 72 h. Full estrous behavior was induced in ovariectomized female rats treated with 5 μg of estradiol benzoate followed by 2 mg of progesterone 44 h later. Under this regime, female rats displayed optimal sexual behavior, characterized by proceptivity and strong lordosis responses (Eguibar et al., 2016). All steroids were purchased from Sigma–Aldrich (St. Louis, MO, USA), diluted in ultra virgin olive oil (Nutrioli, Spain), adjusted to administer a constant volume of 1 mL/kg and injected subcutaneously into the dorsal neck region.

### 2.3 Behavioral testing

Ultrasonic vocalizations were recorded with four ultrasonic microphones with a flat frequency response up to 150 kHz and a working response range from 10 to 180 kHz (Noldus Technologies, The Netherlands). The vocalizations were recorded at a 214 kHz sampling rate and 16-bit depth. The microphones were mounted 7.5 cm above each rat’s compartment, and acoustic insulation was achieved with polyurethane foam. To elicit vocalizations, an estrous-induced female rat approached the incentive zone, which was a circular zone with a 5-cm radius around the sexually experienced male rat of each phenotype, and the USVs were recorded for the following 5 min. The rats were assessed in a four-arm maze (40 × 30 × 45 cm) created with black acrylic plastic with a 4 cm wall thickness and four 25-cm long tubes that were 12 cm in diameter. Each square box (40 × 30 × 30 cm) had a wall with holes to allow visual, hearing and smell senses, but direct contact between the sexes was not permitted (see Fig. 2A). The central square represents a female rat placed with its nose in front of an empty compartment. On the day of the experiment, each male was introduced to a cubic black box for habituation for 10 min before starting the experimental session, and the floor was covered with wood shavings (Aspen Chip, Nepco, USA). The males of different phenotypes were distributed in each square box in an aleatory pattern, and then an estrous-induced ovariectomized female from each phenotype was allowed to freely explore the maze for 5 min. After each test, the maze was cleaned with soap and water and then with 5% alcohol to remove pheromones. Ultrasonic vocalizations were analyzed using Ultra Vox software (Noldus Technologies, The Netherlands) on a computer running Windows 10 software that allows the recording, digitization, and analysis of the USVs and classified as frequency-modulated, flat or trill calls by a trained observer who was unaware of the experimental conditions.

### 2.4 Statistical analysis

The number and latency of the 50 kHz ultrasonic vocalizations and the time spent by females in each male compartment were measured using descriptive statistics and presented as the mean ± standard error of the mean (S.E.M.). Afterward, an analysis of variance (ANOVA) followed by Bonferroni’s multiple range test was applied. The distinct types of calls were evaluated by paired Student’s t tests. For all tests, P ≤ 0.05 was considered to indicate statistical significance. All the statistical analyses were performed using Sigma-Plot software version 14.5 (SYSTAT software, Inc., USA).

## 3 Results

### 3.1 Characterization of ultrasonic calls in high-yawning, low-yawning, and Sprague–Dawley male rats

**Fig. 1:**
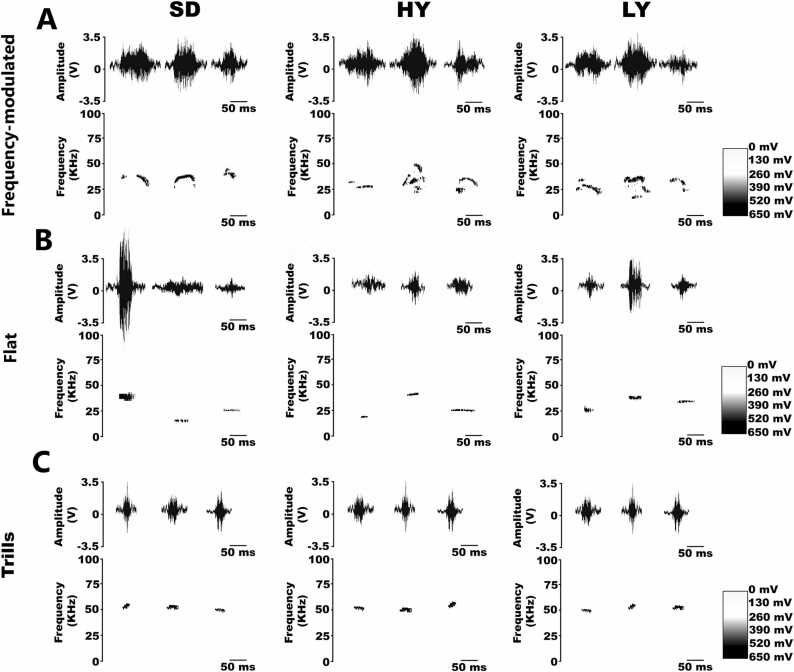
**High-yawning, low-yawning and Sprague-Dawley male rats emit distinct types of ultrasonic vocalizations when they interact with ovariectomized estrous-induced female rats**. Males of the three phenotypes elicited distinct types of ultrasonic vocalizations, named frequency-modulated, flat and trill calls, when they interacted with ovariectomized estrous-induced females in a multichoice maze paradigm. Note that all calls have similar frequency and amplitude characteristics independent of phenotype: high-yawning (HY), low-yawning (LY) or Sprague–Dawley (SD).

When the female approached the goal area in the four-arm maze paradigm, male´s emitted the three types of USVs frequency-modulated, flat or trill calls, suggesting that our multichoice device is functional and is an adequate and reliable method to evaluate USVs. Note that all the male´s emitted frequency-modulated, flat and trills calls with similar frequencies and temporal characteristics (see Fig. 1).

### 3.2 The frequency of the different ultrasonic vocalizations emitted by high-yawning, low-yawning and Sprague–Dawley male rats is dependent on the type of estrous-induced female rat

**Fig. 2:**
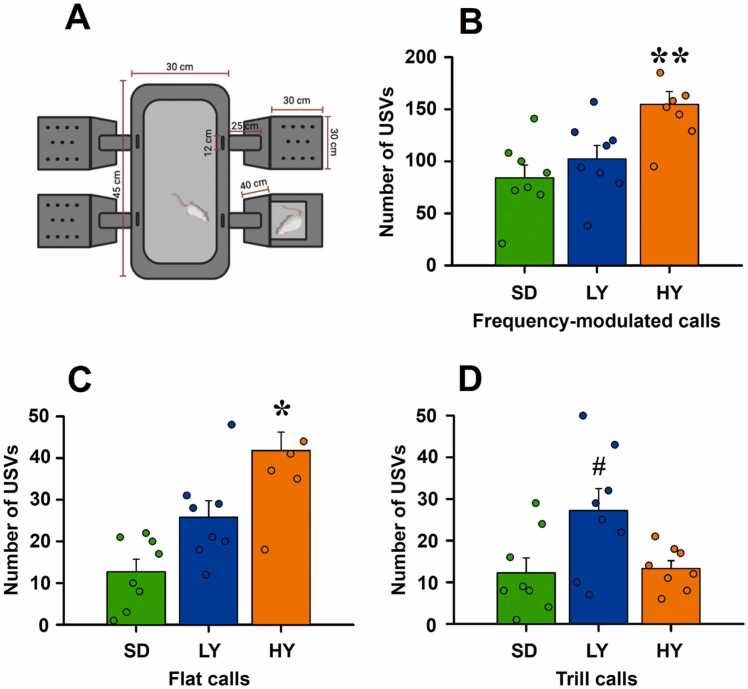
**The number of different ultrasonic vocalizations by high-yawning, low-yawning, or Sprague–Dawley males**. **A)** Experimental setup for recording ultrasonic vocalizations (USVs) during social interaction through a four-arm maze paradigm. Males of each phenotype were placed inside teach grey box, and one arm of the maze remained an empty compartment. The test consisted of placing an ovariectomized estrous–induced female rat primed with estradiol followed by a progesterone in front of the empty compartment and then allowing it to freely explore the maze for 5 min. **B)** High-yawning (HY, orange bars) rats emitted more frequency-modulated (FM) ultrasonic vocalizations than low-yawning (LY, blue bars) or Sprague–Dawley (SD, green bars) male rats did (** P ≤ 0.001). **C)** Compared with SD or LY male rats, high-yawning rats emitted more flat calls (* P ≤ 0.05). **D)** LY male rats emitted more trills than HY or SD males did (# P ≤ 0.05). The data are presented as the mean ± S.E.M. of eight rats of each phenotype.

The number of FM ultrasonic vocalizations differed significantly among the three phenotypes of the rats tested (F_(2)_ = 8.62, P = 0.002), with that of the male HY rats (orange bars) being significantly greater than those of the male SD rats (green bars; t = 4.00, P = 0.002) and the male LY rats (blue bars, t = 2.96, P = 0.02). The number of flat calls also differed among the different groups of male rats (F_(2)_ = 14.94; P = 0.001), with the number of flat calls being significantly greater in the male HY rats with respect to SD rats (t = 5.46; P = 0.001) and the male LY rats (t = 2.99; P = 0.021). Finally, the number of trill calls differed among the different groups of male rats (F_(2)_ = 4.86; P = 0.018), with the number of trill calls being significantly different in male LY rats compared with the male SD rats (t = 2.79; P = 0.033) and male HY rats (t = 2.60; P = 0.05; see Fig. 2).

### 3.3 Time spent in each male compartment by the estrous-induced high-yawning, low-yawning, and Sprague–Dawley female rats

**Fig. 3:**
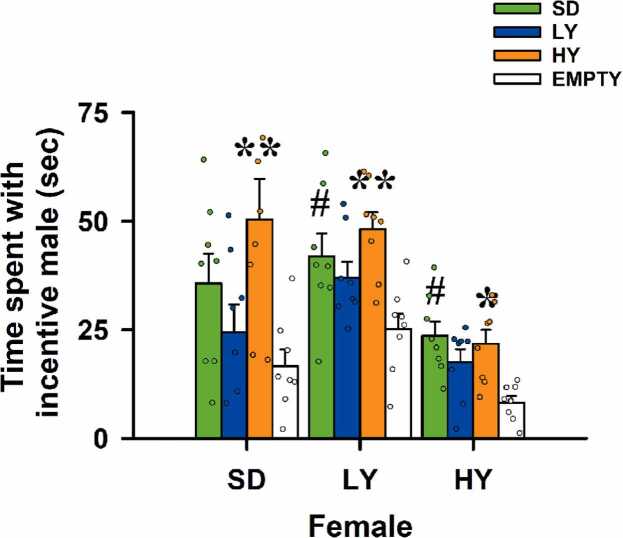
**Time spent by the different female phenotypes in the incentive zone of high-yawning, low-yawning, or Sprague–Dawley males**. The SD (green bars) and HY females (orange bars) preferred the sexually experienced male’s compartment compared with the empty compartment (open bars; H_(3)_= 22.03, P ≤ 0.001, followed by Dunn’s test P ≤ 0.001 and P ≤ 0.002, respectively). SD females preferred to stay close to HY males (t = 3.50, **P ≤ 0.01). The LY (blue bars) females preferred to stay close to the SD males (t = 2.87, # P ≤ 0.05) or the HY males’ incentive zone (t = 3.96, **P ≤ 0.003). HY females preferred to stay in the incentive area of SD males (t = 3.91, # P ≤ 0.05) or that of HY males (t = 3.44, P ≤ 0.05). The data are the mean ± S.E.M. of eight rats of each phenotype.

The time spent in each male compartment by the female rats significantly differed. The estrous females spent more time with the male HY rats (orange bars) (t = 3.50, P = 0.01) than the male SD rats (green bars) (F_(2)_ = 4.61, P = 0.01). The females of different phenotypes spent more time with the male HY rats (t = 3.96, P = 0.003) and male SD rats (t = 2.87, P = 0.046) than the male LY rats (blue bars) (F_(2)_ = 5.062, P = 0.004). When HY males were exposed to the distinct types of female rats (F_(2)_ = 6.10, P = 0.003), HY (t = 3.44, P = 0.05) and SD (t = 3.91, P = 0.05) male rats were the preferred groups of rats (see Fig. 3).

### 3.4 The presence of a receptive female increased the amount of 50-kHz ultrasonic vocalization in high-yawning, low-yawning, and Sprague–Dawley male rats

The frequency-modulated USVs emitted by male Sprague–Dawley rats had a peak frequency of 30.8 ± 0.60 kHz; in male HY rats, the peak frequency was 27.9 ± 0.5 kHz, and in male LY rats, the peak frequency was 27.8 ± 0.9 kHz. These results revealed that for all three male phenotypes, the USVs had similar sonic characteristics. In the case of flat calls, they also had similar frequency characteristics because the peak frequency was 27.8 ± 0.4 kHz in the SD rats: 26.6 ± 1.1 kHz in the HY rats, and 25.3 ± 0.8 kHz in the LY rats.

Male Sprague–Dawley rats had significantly increased numbers of FM calls (Student’s paired t test, t_(14)_ = 3.42; P < 0.05) and trill calls (Student’s t test, t_(14)_ = 3.41; P < 0.05) when they were exposed to a receptive female rat, but this was not the case with flat calls. In the case of LY males, the numbers of FM (Student’s paired t test, t_(14)_ = 6.72, P < 0.01), flat (Student’s paired t test, t_(14)_ = 3.82, P < 0.05), and trill calls (Student’s paired t test, t_(14)_ = 3.82, P < 0.01) significantly increased in the presence of sexually receptive female rats. The frequency of each call type increased when male HY rats were exposed to sexually receptive female rats with the highest number of FM calls among the groups (Student’s paired t test, t_(14)_ = 3.45, P < 0.05; see Table 1). There was also a significant increase in both the number of flat calls (Student’s paired t test, t_(14)_ = 3.38, P < 0.05) and the number of trill calls (Student’s paired t test, t_(14)_ = 2.77, P < 0.05) when exposed to estrous-induced ovariectomized rats.

**Table 1:** Ultrasonic vocalizations of 50 kHz emitted by the different phenotypes of sexually experienced male rats.

	**SD**		**LY**		**HY**	
	**Without a receptive female**	**With a receptive female**	**Without a receptive female**	**With a receptive female**	**Without a receptive female**	**With a receptive female**
**FM**	33.75 ± 8.08	84.25 ± 12.36*	15.12 ± 2.72	102.5 ± 12.71**	83.50 ± 16.57	154.75 ± 12.31*
**Flat**	6.12 ± 0.93	12.75 ± 02.95	09.25 ± 1.98	25.87 ± 03.88*	21.00 ± 04.39	41.87 ± 04.35*
**Trill**	01.75 ± 0.65	12.37 ± 03.47*	04.00 ± 1.07	27.25 ± 05.23**	07.87 ± 0.81	13.37 ± 01.81*

SD: Sprague-Dawley; LY: Low-yawning; HY: High-yawning; FM: Frequency-Modulated.

Student pair t-test, *P < 0.05; **P < 0.001

Data are the mean ± S.E.M. of eight subjects of each phenotype.

## 4 Discussion

The purpose of this study was to analyze the effects induced by a sexually receptive estrous female rat on the USVs emitted by two different male phenotypes of rats that differ in their spontaneous yawning frequency and control Sprague–Dawley male rats. The spectral characteristics of the three types of calls were similar among the three phenotypes analyzed in this study (see Fig. 1), and they had similar characteristics to those already reported in other strains of male laboratory rats (Burgdorf et al., 2011). In the case of 50 kHz, ultrasonic vocalizations play a positive role in providing sexual incentives (Burgdorf et al., 2011) because HY males emitted more calls with shorter latencies and HY males were preferred by ovariectomized estrous-induced female SD and LY rats. Therefore, we concluded that compared with LY and SD rats, high-yawning males were more motivated when exposed to estrous-induced ovariectomized female rats primed with estrogen and progesterone, independent of the female phenotype assessed (see Fig. 2).

Ultrasonic vocalizations represent a critical element in rodent communication. During social encounters, rats emit both 22-kHz and 50-kHz calls depending on the context. Low-frequency 22-kHz calls represent an alarm signal in stressful or threatening situations, whereas higher-frequency 50-kHz vocalizations are strongly associated with positive social interactions such as play, exploration, affiliative contact and even sexual motivation (Heinla et al., 2021). In our multichoice maze paradigm, HY males were clearly more sexually motivated because they emitted a higher number of FM calls.

In fact, using previously recorded 50‑kHz ultrasonic vocalizations and then playback has been revealed to be an adequate appetitive social stimulus. However, in enriched multisensory environments, adult social behavior can proceed normally without vocal output (Tyack, 2008, Heinla et al., 2021). Thus, USVs modulate the affect, expectation, and timing of approach rather than encoding indispensable information for adult social coordination. Developmental stage, arousal, task demands, and experience determine when ultrasonic signals exert measurable control (Brudzynski, 2021, Heinla et al., 2021, Mulvihill and Brudzynski, 2018). In seminatural settings, however, evidence indicates that although USVs accompany many social behaviors, other sensory cues also play a role in guiding social exploration and mating strategies (Heinla et al., 2021, Wöhr and Schwarting, 2013). In fact, devocalization studies suggest that USVs modulate rather than strictly determine adult social interaction patterns (Heinla et al., 2021, Wöhr and Schwarting, 2013). It has been demonstrated that exposure of sexually naïve male rats to an estrous female reliably increases the number of 50-kHz calls, especially the numbers of frequency-modulated and trill calls, such that it is an adequate index of a positive arousal and motivation state. Our results revealed that HY males are more sexually motivated because they emitted the greatest number of FM calls (see Table 1). Additionally, male HY rats were preferred by females with different phenotypes, suggesting that they are sexually attractive and had a stronger motivational state (see Fig. 3). In fact, precontact 50‑kHz USVs emerge with sexual experience and correlate with anticipatory motivation. This phenomenon clearly occurred under our experimental conditions in the four-arm maze test, and they are similar to those obtained under other paradigms (Biały et al., 2000, McGinnis and Vakulenko, 2004). However, multi‑female choice tests revealed that individual differences in female USVs and odors did not predict male partner choice or copulatory outcomes, suggesting that other sensory cues and prior reinforcement dominate mate selection (Snoeren et al., 2014). In our study, SD and LY females preferred HY males, but there was not a sexual interaction. Overall, USVs appear to facilitate approach and arousal under constrained laboratory conditions but are dispensable for mating in naturalistic contexts (Heinla et al., 2021, Snoeren et al., 2014). Our results clearly demonstrated that compared with male SD rats, the males of both yawning sublines were capable of emitting more FM, flat and trill calls (see Table 1), but only HY males were preferred by LY and SD females, suggesting that HY males have greater sexual motivation and are capable of attracting females of different phenotypes through relevant communication pathways, such as ultrasonic vocalizations.

It is well known that the ventral tegmental area–nucleus accumbens pathway is central to appetitive 50‑kHz calling. Phasic dopamine release in the accumbens occurs in listeners during pro‑social 50‑kHz playback, and dopaminergic pharmacology modulates the call rate and microstructure (Willuhn et al., 2014, Brudzynski et al., 2012, Wöhr and Schwarting, 2013, Ringel et al., 2013, Thompson et al., 2006). Systemic injection of D1-receptor antagonists reduces the numbers of amphetamine-evoked and conditioned 50-kHz calls, and D2-receptor mechanisms are involved because quinpirole-induced calling is suppressed by selective antagonists (Brudzynski et al., 2012, Ringel et al., 2013, Thompson et al., 2006). Although one‑to‑one mapping of receptor subtypes to acoustic subtypes remains incomplete, converging evidence indicates that both D1 and D2 families of dopaminergic receptors are necessary to sustain high call rates during appetitive states (Ringel et al., 2013, Thompson et al., 2006). It is relevant that across social and nonsocial appetitive contexts, haloperidol broadly blunts 50‑kHz emission, underscoring the dependence of appetitive vocalization on intact mesolimbic dopamine signaling (Mulvihill and Brudzynski, 2018, Ringel et al., 2013). Compared with adult male LY rats, adult male HY rats exhibit higher frequencies of yawning after systemic injection of different dopaminergic D2 agonists, such as apomorphine or quinpirole (Eguibar et al., 2003, Urbá-Holmgren et al., 1993). Therefore, differences in dopaminergic tone could be the cause of distinct types of calls among yawning sublines and with respect to male Sprague–Dawley rats. Our results clearly revealed that high-yawning males had greater arousal and motivation when they expressed more USVs with shorter latencies and were exposed to females with different phenotypes in a multiple sexual choice paradigm, supporting the notion that male HY rats exhibit a higher motivation state.

## 5 Conclusion

In summary, the results presented here indicate that high-yawning males emitted more 50-kHz ultrasonic vocalizations and that LY and SD females preferred to stay close to sexually experienced HY male rats, suggesting that HY males exhibit enhanced arousability and sexual motivation under a multichoice paradigm.

## CRediT authorship contribution statement

**Alfonso Mora:** Validation, Software, Methodology, Investigation, Formal analysis, Data curation. **Juan M. Ibarra-Hernandez:** Visualization, Validation, Methodology, Investigation, Formal analysis, Data curation. **Jose R. Eguibar:** Writing – review & editing, Writing – original draft, Validation, Supervision, Resources, Project administration, Methodology, Investigation, Funding acquisition, Formal analysis, Data curation, Conceptualization. **Araceli Ugarte:** Supervision, Methodology, Formal analysis. **Carmen Cortes:** Writing – review & editing, Writing – original draft, Validation, Supervision, Project administration, Investigation, Funding acquisition, Formal analysis, Data curation, Conceptualization.

## Ethical standards

All procedures described in this study were performed in compliance with the Mexican official standard technical specifications for the production, care and use of laboratory animals (NOM-062-ZOO-1999), and with the Seventh Title of the Regulations of the General Health Law on Health Research of the Mexican government, as well as the Guide for the Care and Use of Laboratory Animals of the Institute for Laboratory Animal Research and the National Research Council of the National Academies (National Research Council, 2011). The ARRIVE guidelines 2.0 for animal research were followed throughout the study (Percie du Sert et al., 2020). The Institutional Committee for the Care and Use of Laboratory Animals of the Benemérita Universidad Autónoma de Puebla (CICUAL-Proyecto BUAP- 15755) approved all animal procedures.

## Funding

This research was supported by Vicerrectoría de Investigación y Estudios de Posgrado, Benemérita Universidad Autónoma de Puebla 2025 to Cuerpo Académico en Neuroendocrinología (BUAP-CA-288). AMB received a fellowship from SECIHTI for his Ph.D. studies in Physiological Sciences with No. 662091. We thank American Journal Experts for editing the English-language text verification code 475F-0EC1-DACC-0AA5–53E7.

## Declaration of Competing Interest

The authors declare no conflicts of interest concerning this research. Funding had no role in the design, analyses, or interpretation of the data in the study.

## Data Availability

Data that supports the findings of this study can be obtained from the corresponding authors upon reasonable request.
